# Bridging a translational gap: using machine learning to improve the prediction of PTSD

**DOI:** 10.1186/s12888-015-0399-8

**Published:** 2015-03-16

**Authors:** Karen-Inge Karstoft, Isaac R Galatzer-Levy, Alexander Statnikov, Zhiguo Li, Arieh Y Shalev, For members of the Jerusalem Trauma Outreach and Prevention Study (J-TOPS) group

**Affiliations:** 1 Department of Psychiatry, NYU School of Medicine, New York, NY USA; 2 Research and Knowledge Centre, Danish Veteran Centre, Garnisonen 1, 4100 Ringsted, Denmark; 3 Center for Health Informatics and Bioinformatics, NYU School of Medicine, New York, NY USA; 4 Department of Medicine, NYU School of Medicine, New York, NY USA

**Keywords:** Posttraumatic Stress Disorder (PTSD), Machine learning, Early prediction, Risk factors, Markov boundary feature selection, Support vector machines

## Abstract

**Background:**

Predicting Posttraumatic Stress Disorder (PTSD) is a pre-requisite for targeted prevention. Current research has identified group-level risk-indicators, many of which (e.g., head trauma, receiving opiates) concern but a subset of survivors. Identifying interchangeable sets of risk indicators may increase the efficiency of early risk assessment. The study goal is to use supervised machine learning (ML) to uncover interchangeable, maximally predictive combinations of early risk indicators.

**Methods:**

Data variables (features) reflecting event characteristics, emergency department (ED) records and early symptoms were collected in 957 trauma survivors within ten days of ED admission, and used to predict PTSD symptom trajectories during the following fifteen months. A Target Information Equivalence Algorithm (TIE*) identified all minimal sets of features (Markov Boundaries; MBs) that maximized the prediction of a non-remitting PTSD symptom trajectory when integrated in a support vector machine (SVM). The predictive accuracy of each set of predictors was evaluated in a repeated 10-fold cross-validation and expressed as average area under the Receiver Operating Characteristics curve (AUC) for all validation trials.

**Results:**

The average number of MBs per cross validation was 800. MBs’ mean AUC was 0.75 (95% range: 0.67-0.80). The average number of features per MB was 18 (range: 12–32) with 13 features present in over 75% of the sets.

**Conclusions:**

Our findings support the hypothesized existence of multiple and interchangeable sets of risk indicators that equally and exhaustively predict non-remitting PTSD. ML’s ability to increase prediction versatility is a promising step towards developing algorithmic, knowledge-based, personalized prediction of post-traumatic psychopathology.

**Electronic supplementary material:**

The online version of this article (doi:10.1186/s12888-015-0399-8) contains supplementary material, which is available to authorized users.

## Background

The early identification of individuals at risk for developing posttraumatic stress disorder (PTSD) is a major clinical and public health challenge, which many studies have attempted to address (for meta-analyses, see Brewin et al. [1] and Ozer *et al.* [2]). Currently-identified risk indicators include event characteristics [3], peri-traumatic responses [4-6], early symptoms [7-10], early physiological and neuroendocrine responses [11], gene expression profiles [12] and recovery environment factors [13]. Together, current findings suggest that PTSD is associated with an array of multimodal risk indicators, many of which are observable shortly after trauma exposure. Despite these findings, research to date has failed to reveal clinically useful, personalized predictors.

This translational gap has several reasons: Previous studies have identified risk indicators at the group level, thereby overlooking within-group heterogeneities and distinct individual paths to PTSD that emanate from the disorder’s complex multi-causal etiology [14]. Based on the general linear model, statistical methods used were not optimally suited to explore the complex interactions between linear, non-linear or non-normally distributed risk indicators encountered during trauma and its early aftermath [15].

Additionally, within the inherently complex and multimodal matrix of emerging post-traumatic morbidity, the relative contribution of any risk-indicator is necessarily context-dependent and thus does not directly translate across traumatic events and individuals exposed (e.g., female gender increases the likelihood of PTSD among survivors of physical assault, but not in accidents victims [3]). Consequently proper risk assessment defies simple computation and requires knowledge-based, rule driven expert systems.

Importantly, many of the currently known risk indicators may not be present, or not captured in every exposed individual. For example, elevated heart rate response to traumatic events, whilst repeatedly associated with subsequent PTSD [5,16] is only recorded in survivors who are brought to medical attention. Other known risk moderators, such as head injury [17,18] or receiving opiates following injury [19] similarly concern a subset of survivors.

To overcome these limitations, forecasting methods of PTSD must accommodate multiple combinations of risk indicators, account for partially available information and use prior knowledge to adjust the relative weights of putative predictors. The goal of the present work is to address the first requirement, namely, evaluate the use of multiple combinations or ‘sets’ of data items to predict post-traumatic morbidity, assess the accuracy the predictive power made from such sets.

To accomplish this goal, this work applied machine learning (ML, see glossary in Additional file 1) modeling to a large longitudinal dataset and evaluated the method’s ability to identify multiple, equally predictive sets of variables. ML based forecasting models can accommodate different configurations of predictive features, integrate multi-modal variables, assign context-driven weights to putative predictors and identify multiple sets of variables that exhaust the predictive power of available features [20-22].

In a previous study [23], we evaluated the ability of ML-based *feature-selection* algorithm to extract one set of early risk indicators. We also compared various ML classification algorithms and evaluated predictability of two outcome configurations: PTSD at end point and membership in a non-remitting PTSD symptom trajectory. That study demonstrated that data representing the traumatic event and subsequent ED admission (e.g., head injury, length of stay in the ED) improves the prediction from early symptoms. It showed equal performance of six classification algorithms and better predictability of the non-remitting PTSD symptom trajectory relative to diagnostic status. Building on these findings, this work uses support vector machines (SVMs) as its classification algorithm and a non-remitting PTSD symptom trajectory as the predicted outcome. This work, therefore, expands the scope of the previous study by evaluating multiple, equivalent, maximally predictive sets of early features.

**Figure 1:**
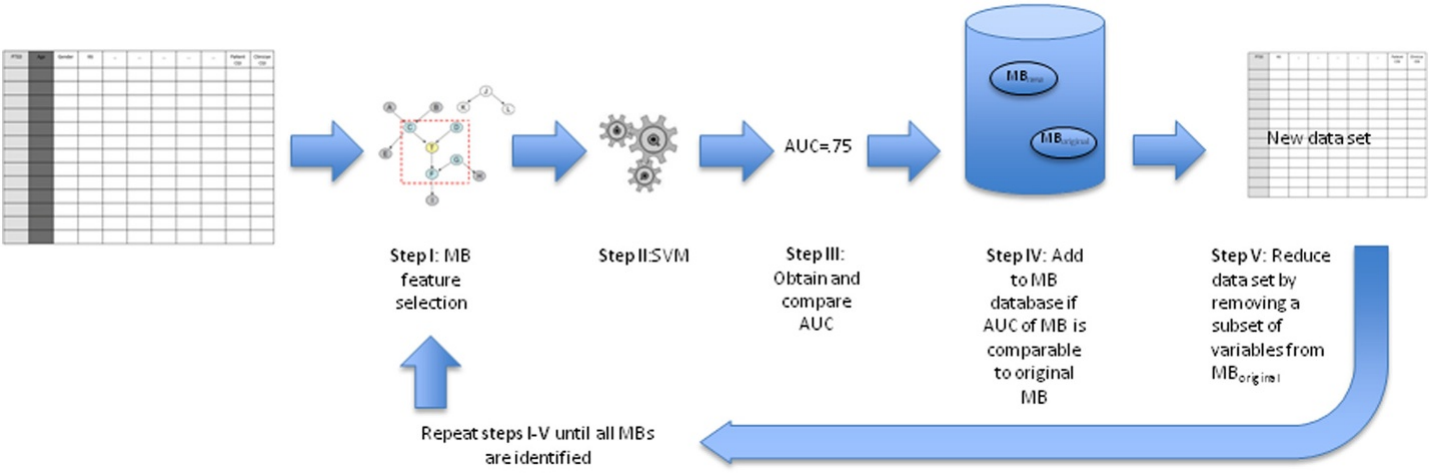
TIE* Algorithm Flow Chart. The figure outlines the successive steps used by the TIE* algorithm to, first, identify (step I) and validate (step II) compact set of maximally predictive risk indicators (MBs), calculate ROC curve AUC for the MB (step III), include the MB in a pool of MBs if AUC ≥ that for the original MB (Step IV), extract MB features from the dataset (step V) and reiterate steps I to V until all MBs in a dataset are identified.

Specifically, we applied a Target Information Equivalence algorithm (TIE-star or TIE*; Figure 1) to uncover *all* compact non-redundant sets of items that maximize the prediction of non-remitting PTSD symptom trajectory [20,4]. We then evaluated the accuracy of prediction from each of these sets using support vector machines (SVMs [24]).

## Methods

### Participants and Procedures

This study used data collected for the Jerusalem Trauma Outreach and Prevention Study (J-TOPS [25-27]; ClinicalTrial.Gov identifier: NCT0014690). Participants were adults (age: 18–70) consecutively admitted to ED following potentially traumatic events (PTEs). Participants provided oral and written informed consent for, respectively, telephone based and face-to-face phases of the study. The study’s procedures were approved and monitored by the Hadassah University Hospital’s Institutional Review Board.

Eligible participants (n = 4,743) were screened by short telephone interviews, and those with confirmed PTEs as per DSM-IV PTSD criteria A1 and A2 (n = 1,996) received structured, telephone-based interviews ten days (exactly 9.61 ± 3.91 days) after trauma exposure. Telephone based assessments were repeated seven months (n = 1,784) and fifteen months (n = 1,022) following ED admission. Participants with acute PTSD symptoms in the first assessment (n = 1,502) were additionally invited for clinical interviews, which n = 756 attended, 19.80 ± 5.17 days after ED admission. Participants of the first clinical assessment were re-evaluated five months after the traumatic event (144.1 ± 35.2 days; n = 604). For detailed procedures, see Shalev et al. [27].

For the purpose of this study, we included individuals who had initial data available at ten days and at least two additional time points (n = 957). Participants included in this study did not differ from those who were not included in gender distribution, age, general distress, initial PTSD symptoms and the frequency of exposure to new traumatic events during the study [24].

### Instruments

Sixty-eight data items (features) were recorded during survivors’ ED admission and in the first telephone interviews. *ED features* included demographics, trauma types (traffic accidents, work accidents, terrorist attacks and other incidents), loss of consciousness during the traumatic incident, head injury, whiplash injury, blood pressure, pulse, perceived pain (a 1–10 pain intensity scale), prescribed analgesics and duration of ED admissions. *Telephone interview features* included individual items and total scores of the PTSD symptom scale (PSS) [28], the Kessler-6 (K6), [29] the Acute Stress Disorder Scale (ASDS) [30] and the Clinical Global Impression instrument (CGI) [31] which both participants and interviewers completed. They also included single items reflecting the four dimensions of the Posttraumatic Cognition Inventory (PTCI [32]: (counting on others, counting on oneself, dangerousness of the world, and self-blame) and four coping efficacy items [33] (sustained task performance, capacity for rewarding interpersonal communication, controllability of emotions, and positive self-perception), as well as participants’ expressed need for help, perceived social support, and perceived fearfulness and threat embedded in the traumatic event.

### Modeling Approach

#### Outcome Measure

The study’s main outcome measure was membership (yes/no) in a non-remitting PTSD symptom trajectory as defined in a previous LGMM-based study of this sample [25]. Studies have shown that the alternative outcome, PTSD diagnostic status, is unstable, fluctuates with time [34] and can be met with various degrees of symptom severity. In this dataset, the non-remitting PTSD symptom trajectory was not affected by treatment received and, as mentioned above, was better predicted than end point PTSD status [23].

#### Machine learning approach

##### Identification of risk indicators sets (MBs, Figure 1)

To identify all compact sets of variables with optimal predictive accuracy, we applied the TIE* (Target Information Equivalence - Star) algorithm [20]. The TIE* algorithm: (i) Identifies a minimal set of variables that render all other predictors non-significant in relation to the outcome (a ‘Markov Boundary’ or, MB) and evaluates the accuracy of prediction using SVM, (ii) removes one or more of the obtained MB features from the data set, and repeats the analyses to identify a new MB in the reduced data set, (iii) determines the accuracy of the new MB by feeding it in to a Support Vector Machine, and keeps the new MB if its predictive accuracy is statistically comparable to that of the original MB, and (iv) reiterates steps (ii) and (iii) until *all MBs that provide equivalent predictive accuracy have been identified* (Figure 1). The TIE* has been validated in previous studies [20]. The MB identification processes used in the TIE* are available in the Causal Explorer toolkit [35]. The SVM algorithm used is available at LibSVM [36].

##### Cross-validation procedure

To test the robustness of predictors, we subsequently applied the TIE* procedure in a 10-fold cross-validation, in which participants are randomly split into ten non-overlapping subsets containing approximately the same number of cases and non-cases (patients following a non-remitting and a remitting course of PTSD, respectively). The classification algorithm is trained in nine of these ten data subsets, and subsequently (and independently) tested in the remaining tenth subset. This procedure is repeated iteratively, resulting in each of the ten data subsets being used once for testing of the model. We repeated the 10-fold cross validation procedure 10 times to reduce splitting variance, resulting in a total of 100 repetitions of training and testing. Predictive accuracy was expressed as the mean accuracy obtained from SVM applied across all cross-validation runs. The frequency of features’ presence across MBs was calculated as a measure of their predictive ‘robustness’.

### Accuracy metric

We estimated predictive accuracy using Area Under the ROC Curve (AUC). The ROC curve is a plot of the sensitivity versus 1-specificity of a classification system, and measures the accuracy of that system, which can then be directly compared to that of another system [37]. To further investigate the accuracy, we also computed average sensitivity and specificity for various thresholds.

## Results

### Identification of MBs in full data set

Before cross-validation, the TIE* algorithm applied to the full data set (N = 957) identified 789 distinct MBs. The average number of data items per MB was 18 (range 15–29). Thirty-four items participated in at least one MB.

### Cross-validation of MBs

**Figure 2:**
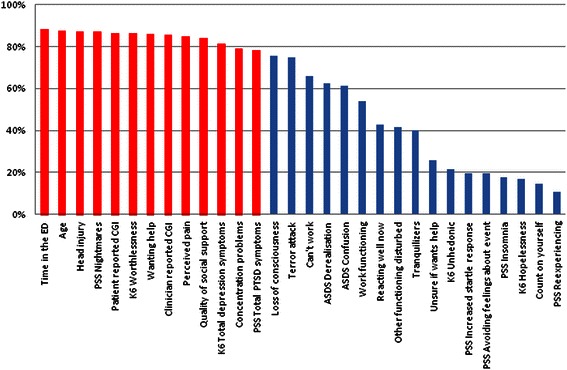
**Feature’s presence in repeated cross validation trials.** The figure shows the frequency (percentage of all trials) in which individual features participate in MBs identified during successive cross-validations trials (only features participating in >10% of the trials are presented). Bars in red indicate features selected in >75% of cross validation runs (n = 13).

The average number of MBs identified in the repeated cross-validations was 800. The average number of features per MB was 18 (range 12–32). Forty-seven features participated in at least one MB. Thirteen features participate in over 75% of all MBs (see Figure 2). The consistently predictive features include *age, time in the ED, head injury, perceived ED pain, patient and clinician’s clinical global impression, total PSS and K6 scores, reporting nightmares, concentration problems, feeling worthless, wanting help, and quality of social support*. The average predictive accuracy of all MBs was within an acceptable range (AUC = .75; 95% range = 0.67 - 0.80).

## Discussion

The study’s findings support the hypothesized existence of multiple interchangeable combinations of risk indicators that equally predict non-recovery from information obtained within days of a traumatic event. Specifically, before cross validation, we identified 789 minimal sets of variables (MBs) that rendered all others non-significant predictors of non-remitting PTSD. The average number of MBs per cross validation trial was 800. This minor difference is expected, given use of slightly different datasets (i.e., total dataset vs. randomly selected sets of 90% of the observations).

The existence of such large number of MBs may reflect the presumed multi-causal and equifinal etiology of post-traumatic morbidity, which posits many interchangeable contributing factors and many causal pathways. It is also in line with prior evidence of multiplicity of distinct risk indicators of PTSD [7,12,38].

Our finding extends previous work by translating the previously demonstrated multiplicity of risk indicators into versatile predictive model that can accommodate an array of traumatic situations where one or several known predictors is either unavailable or not contributing significantly. From a practical point of view, such multiplicity points to the potential usefulness of data-informed algorithmic prediction tools to future risk assessments.

This work also extends the array of risk indicators identified by earlier studies: Former studies uncovered salient predictors within large groups, whereas this work demonstrated the ability of less consistently predictive, or less frequently recorded features (e.g., expressing a need for help, or ED length of stay) to carry important information. This underscores ML ability to *not to reject features that are only weakly, or occasionally correlated with an outcome*, and thereby fully extract the informational item of datasets.

Within such multiplicity, however, this study identified a few consistently predictive features (e.g., those included in over 75% of all MBs). Interestingly, these features comprised, side-by-side, prior variables (e.g., age), event and injury parameters, immediate bodily responses (e.g., ED pain), symptoms (nightmares, loss of concentration, total PTSD and depression symptoms), clinicians’ observations (e.g., CGI) and more elaborated subjective responses (need for help, sense of worthlessness). Surprisingly, gender was not among the consistent predictors. This might reflect the nature of traumatic events evaluated for this study, most of which were road traffic accidents and thus not gender specific.

MB’s predictive accuracy (AUC = .75) does not support a robust prediction from early information collected. This may illustrate the limited predictive power of data features available for this study, all collected within ten days of a traumatic event. Within such limitations, the results of this work still fare well on two accounts: They firstly show the already reasonable ability of simple, non-invasive, inexpensive observations to predict post-traumatic morbidity. They additionally establish the usefulness of data features that are regularly collected in ED situations. Indeed, this work was not meant to show superior predictive performance, but rather to establish, within the predictive power of a dataset, ways to increase predictive versatility.

Predicting from very early features is also limited in that early symptoms are ubiquitous whereas subsequent morbidity is less frequent (17% non-remitting in this work). Previous work has shown better prediction of chronic PTSD by data collected one month after the traumatic event [39]. Moreover, other known risk indicators that might become available within days of traumatic exposure (e.g., childhood trauma [40], lifetime mental disorders [41], ED stress hormones [42], gene variants (e.g., FKBP5 [43]), or ED gene expression profiles [12,44]) have not been assessed in this study. Expanding the array of early predictive features by collecting such data might improve the predictive accuracy of early observations.

An important remaining question is the added contribution of putative biomarkers to prediction from of non-invasive, easily retrievable clinical data: Clinical manifestations may express the compounded effect of underlying biological vulnerability and thus might constitute more easily obtainable, non-invasive proxy variables of the latter. Because ML methods can accommodate multimodal information they might help establishing such ‘proxy’ relationships.

**Figure 3:**
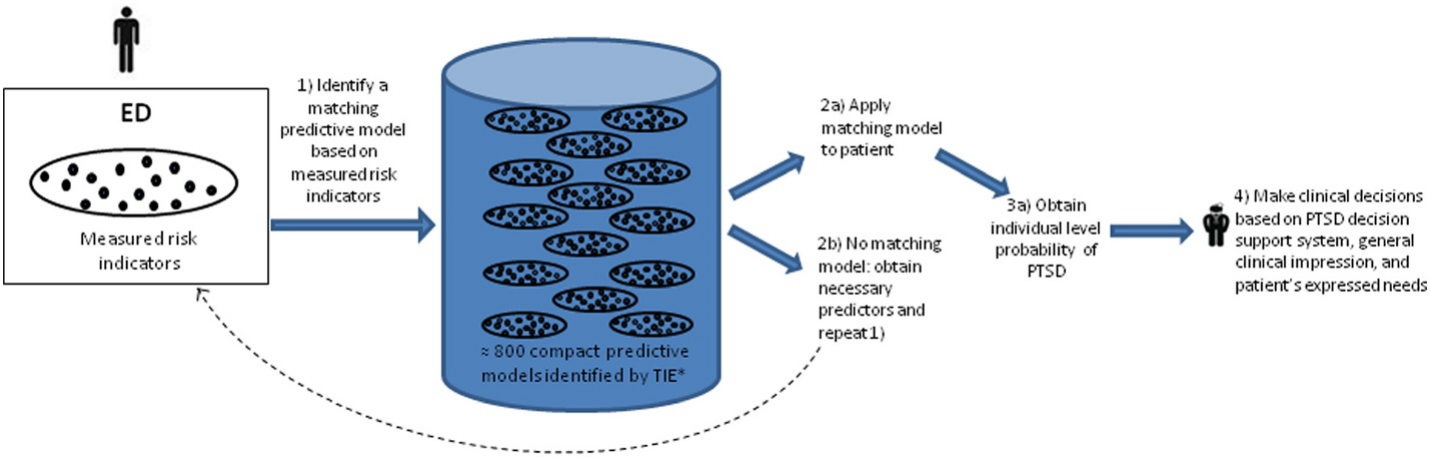
**Data-Informed Decision Support Tool to Forecast PTSD.** This figure outlines a scenario for future implementation of multiple predictive models within a decision support tool for estimating the individual risk. A patient is admitted to the ED after exposure to a potentially traumatic event and a range of risk indicators are assessed. From the collection of models previously identified, in this and subsequent studies, a best matching set of risk indicators is identified (step 1) and, if needed, the system prompts the clinician to seek information about missing risk indicators. Once enough data is available (step 2) a matching model is applied and personal risk estimate computed (step 3).

Our results are far from exhausting the potential of machine learning to forecast PTSD. Following similar progress in other areas of medicine [45], ML approaches for forecasting post-traumatic morbidity must be extended and enriched using other data sets and adding other putative predictors. One of many scenarios of such future use of ML decision support algorithms is illustrated in Figure 3. The figure shows how cumulative knowledge of predictive MBs can progressively enrich knowledge-informed algorithmic approach for risk assessment.

## Conclusions

By providing greater versatility, ML-informed algorithms may better identify individuals at risk for post-traumatic morbidity under varying traumatic circumstances. ML capacity to accommodate multimodal information offers new heuristic for forecasting post-traumatic morbidity.

## Additional file

Additional file 1:
**Glossary.**
