# Supplementary material for: Bridging a translational gap: using machine learning to improve the prediction of PTSD
Source: BMC Psychiatry. 2015 Mar 16;15:30. doi: 10.1186/s12888-015-0399-8 (PMC4360940; doi:10.1186/s12888-015-0399-8)
Supplement: Additional file 1: — Glossary. [file 12888_2015_399_MOESM1_ESM.docx]

**GLOSSARY & ABBREVIATIONS**

- **AUC** – Area under ROC curve.
- **MB -** Markov Boundary, a non-redundant set of variables that leaves all other variables independent of the target when conditioning on that set
- **Feature (or variable)** – a descriptor of a characteristic recorded for each participant. For example: age, a specific PTSD symptom, head injury, blood pressure, recorded for each patient in a cohort.
- **ML** – Machine Learning; a subfield of computer science concerned with the study and construction of systems that learn from data
- **Outcome (or target, or dependent) variable** – A variable that researchers are interested in predicting as a function of predictor variables.
- **Predictor variable (or predictor, or independent variable)** – A variable used for prediction/modeling of an outcome (ie, response) variable, alone or in combination with other predictors.
- **ROC curve** – Receiver Operating Characteristic curve.
- **Supervised Machine Learning** – the building of a predictor model based on known input data and known responses to the data
- **SVM** – Support Vector Machines. A supervised machine learning approach that categorizes individuals into known classes by identification of a hyperplane in high-dimensional space
- **Testing set** – portion of the data (random subset) used to estimate how well a previously trained and validated model will perform in future independent samples from the same population.
- **TIE*** - Target Information Equivalence, a machine learning algorithm for identification of multiple (all) MBs in a data set.
- **Training set/data/dataset/sample** – portion of the data (subset of samples) used to develop (ie, “train” or fit parameter values for) a classification or regression algorithm.
